# [Retracted] SIRT1 promotes tumorigenesis of hepatocellular carcinoma through PI3K/PTEN/AKT signaling

**DOI:** 10.3892/or.2023.8547

**Published:** 2023-04-18

**Authors:** Hanning Wang, Hao Liu, Kaiyun Chen, Jinfeng Xiao, Ke He, Jinqian Zhang, Guoan Xiang

Oncol Rep 28: 311–318, 2012; DOI: 10.3892/or.2012.1788

Following the publication of this paper, it was drawn to the Editor's attention by a concerned reader that the control β-actin western blots shown in Figs. 1B and 6 were strikingly similar to data that had already appeared in a different form in the following publication: Lei Y, Liu H, Yang Y, Wang X, Ren N, Li B, Liu S, Cheng J, Fu X and Zhang J: Interaction of LHBs with C53 promotes hepatocyte mitotic entry: A novel mechanism for HBV-induced hepatocellular carcinoma. Oncol Rep 29: 151–159, 2012.

Owing to the fact that the contentious data in the above article had already been published prior to its submission to *Oncology Reports*, the Editor has decided that this paper should be retracted from the Journal. After having been in contact with the authors, they accepted the decision to retract the paper. The Editor apologizes to the readership for any inconvenience caused.

